# Children with hepatitis B virus infections, Democratic Republic of the Congo

**DOI:** 10.2471/BLT.24.292013

**Published:** 2025-04-08

**Authors:** Camille E Morgan, Kimberley A Powers, Jess K Edwards, Upasana Devkota, Stane Biju, Feng-Chang Lin, John L Schmitz, Gavin Cloherty, Jérémie Muwonga, Aimée Mboyo, Pascal Tshiamala, Melchior M Kashamuka, Antoinette Tshefu, Michael Emch, Marcel Yotebieng, Sylvia Becker-Dreps, Jonathan B Parr, Peyton Thompson

**Affiliations:** a Department of Epidemiology, Gillings School of Global Public Health, University of North Carolina, 111 Mason Farm Rd, Chapel Hill, NC 27599, United States of America (USA).; b Infectious Disease Epidemiology and Ecology Lab, University of North Carolina, Chapel Hill, USA.; c Department of Biostatistics, Gillings School of Global Public Health, University of North Carolina, Chapel Hill, USA.; d Department of Pathology and Laboratory Medicine, University of North Carolina School of Medicine, Chapel Hill, USA.; e Abbott Laboratories, Abbott, USA.; f Programme National de Lutte Contre le SIDA, Kinshasa, Democratic Republic of the Congo.; g National Hepatitis Control Program, Kinshasa, Democratic Republic of the Congo.; h École de Santé Publique, Université de Kinshasa, Kinshasa, Democratic Republic of the Congo.; i Department of Medicine, Albert Einstein College of Medicine, Bronx, USA.; j Department of Family Medicine, University of North Carolina School of Medicine, Chapel Hill, USA.; k Department of Medicine, University of North Carolina School of Medicine, Chapel Hill, USA.; l Department of Pediatrics, University of North Carolina School of Medicine, Chapel Hill, USA.

## Abstract

**Objective:**

To characterize childhood hepatitis B virus (HBV) epidemiology to inform elimination efforts in the Democratic Republic of the Congo, one of the most populous African countries.

**Methods:**

Using the most recent (2013–2014) nationally representative Demographic and Health Survey, we analysed hepatitis B surface antigen (HBsAg) on dried blood spots and associated survey data from children aged 6–59 months. We estimated HBsAg-positivity prevalence nationally, regionally and by potential correlates of infection. We evaluated spatial variation in HBsAg-positivity prevalence overall, and by age, sex and vaccination status.

**Findings:**

Using data representing 5773 children, we observed a national HBsAg-positivity prevalence of 1.3% (73/5773; 95% confidence interval, CI: 0.9 to 1.7), ranging from 0.0% in Kinshasa to 5.6% in Sud-Ubangi. Prevalence among boys (1.8%; 95% CI: 1.2 to 2.7) was double that among girls (0.7%; 95% CI: 0.4 to 1.3). Testing negative for tetanus antibodies, rural residence and poorer household were associated with higher HBsAg-positivity prevalence. We observed no difference in prevalence by age. Children had higher HBsAg-positivity odds if living with one or more HBsAg-positive adult household member (odds ratio, OR: 2.3; 95% CI: 0.7 to 7.8), particularly an HBsAg-positive mother (OR: 7.2; 95% CI: 1.6 to 32.3). Notably, nearly two thirds (36/51) of HBsAg-positive children had a HBsAg-negative mother.

**Conclusion:**

Our investigation highlights the importance of subnational prevalence estimates in large countries such as the Democratic Republic of the Congo, and we have identified regions that may benefit from improved childhood vaccination delivery strategies and community HBV prevention efforts.

## Résumé

**Objectif:**

Caractériser l’épidémiologie du virus de l’hépatite B (VHB) chez l’enfant pour informer les efforts d’élimination en République démocratique du Congo, l’un des pays africains les plus peuplés.

**Méthodes:**

En utilisant l’Enquête démographique et de santé la plus récente (2013–2014) et représentative à l’échelle du pays, nous avons analysé l’antigène de surface de l’hépatite B (HBsAg) sur des taches de sang séché et des données d’enquête associées concernant des enfants âgés de 6 à 59 mois. Nous avons estimé la prévalence de la positivité à l’HBsAg au niveau national, régional et en fonction des corrélats potentiels de l’infection. Nous avons évalué les variations spatiales de la prévalence de positivité à l’HBsAg dans son ensemble, ainsi qu’en fonction de l’âge, du sexe et du statut vaccinal.

**Résultats:**

L’utilisation de données totalisant 5773 enfants nous a permis d’observer une prévalence nationale de l’HBsAg de 1,3% (73/5773; intervalle de confiance (IC) à 95%: 0,9–1,7), allant de 0,0% à Kinshasa à 5,6% dans le Sud-Ubangi. La prévalence chez les garçons (1,8%; IC à 95%: 1,2–2,7) était deux fois plus élevée que chez les filles (0,7%; IC à 95%: 0,4–1,3). Un test négatif pour les anticorps antitétaniques, un lieu de résidence en milieu rural et un ménage plus pauvre étaient associés à une prévalence plus élevée de la positivité à l’HBsAg. Nous n’avons observé aucune différence de prévalence en fonction de l’âge. Les enfants étaient davantage susceptibles d’être positifs à l’HBsAg s’ils vivaient avec un ou plusieurs membres adultes du ménage eux aussi positifs à l’HBsAg (odds ratio (OR): 2,3; IC à 95%: 0,7–7,8), en particulier avec une mère positive à l’HBsAg (OR: 7,2; IC à 95%: 1,6–32,3). Notamment, près des deux tiers (36/51) des enfants positifs à l’HBsAg avaient une mère négative à l’HBsAg.

**Conclusion:**

Notre enquête souligne l’importance des estimations de prévalence infranationales dans les pays de grande taille comme la République démocratique du Congo, et nous avons identifié des régions susceptibles de bénéficier de meilleures stratégies de vaccination des enfants et d’efforts de prévention contre le VHB à l’échelle communautaire.

## Resumen

**Objetivo:**

Caracterizar la epidemiología infantil del virus de la hepatitis B (VHB) para orientar los esfuerzos de eliminación en la República Democrática del Congo, uno de los países más poblados de África.

**Métodos:**

Se utilizaron los datos más recientes disponibles de la Encuesta Demográfica y de Salud representativa a nivel nacional (2013-2014), en los que se analizaron muestras de sangre seca para detectar el antígeno de superficie del virus de la hepatitis B (HBsAg) y se integraron con los datos de la encuesta correspondiente a menores de entre 6 y 59 meses. Se estimó la prevalencia de positividad al HBsAg a nivel nacional, regional y según posibles factores correlacionados con la infección. Asimismo, se evaluaron las variaciones espaciales de la prevalencia de positividad al HBsAg de forma general y según edad, sexo y estado vacunal.

**Resultados:**

Con datos representativos de 5773 menores, se observó una prevalencia nacional de positividad al HBsAg del 1,3% (73/5773; intervalo de confianza [IC] del 95%: 0,9 a 1,7), con un rango que iba del 0,0% en Kinsasa al 5,6% en Sud-Ubangi. La prevalencia en los niños varones (1,8%; IC del 95%: 1,2 a 2,7) fue el doble que en las niñas (0,7%; IC del 95%: 0,4 a 1,3). Se observó una mayor prevalencia de positividad al HBsAg entre quienes presentaban seronegatividad frente al tétanos, residían en zonas rurales o pertenecían a hogares con menor nivel socioeconómico. No se identificaron diferencias de prevalencia según la edad. Las probabilidades de positividad al HBsAg fueron más elevadas entre menores que convivían con al menos un adulto del hogar positivo para HBsAg (razón de posibilidades [OR]: 2,3; IC del 95%: 0,7 a 7,8), especialmente si se trataba de una madre positiva para HBsAg (OR: 7,2; IC del 95%: 1,6 a 32,3). Cabe destacar que casi dos tercios (36/51) de los menores positivos para HBsAg tenían madres negativas para este marcador.

**Conclusión:**

Esta investigación destaca la importancia de contar con estimaciones de prevalencia a nivel subnacional en países de gran tamaño, como la República Democrática del Congo, e identifica regiones que podrían beneficiarse de estrategias mejoradas para la vacunación infantil y de intervenciones comunitarias para la prevención del VHB.

## ملخص

الغرض توصيف وباء فيروس التهاب الكبد الوبائي ب (HBV) لدى الأطفال لتوجيه جهود القضاء عليه في جمهورية الكونغو الديمقراطية، إحدى أكثر الدول الأفريقية ازدحامًا بالسكان.

المشكلة باستخدام أحدث مسح سكاني صحي وطني (2013-2014) ممثلاً للصعيد الوطني، قمنا بتحليل المستضد السطح لالتهاب الكبد الوبائي ب (HBsAg) في بقع الدم الجافة، وبيانات المسح المرتبطة به من الأطفال الذين تتراوح أعمارهم بين 6 و59 شهرًا. قمنا تقدير معدل انتشار إيجابية مستضد التهاب الكبد الوبائي ب على الصعيدين الوطني والإقليمي، وحسب العوامل المرتبطة المحتملة بالإصابة. كما قمنا بتقييم التباين المكاني في معدل انتشار إيجابية مستضد التهاب الكبد الوبائي ب بشكل عام، وحسب العمر، والجنس، وحالة التطعيم.

النتائج باستخدام بيانات تمثل 5773 طفلاً، لاحظنا معدل انتشار إيجابية المستضد HBsAg على المستوى الوطني بنسبة %1.3 (73/5773؛ بفاصل ثقة مقداره %95: 0.9 إلى 1.7)، يتراوح من %0.0 في كينشاسا إلى %5.6 في سود أوبانجي. كان معدل الانتشار بين الأولاد (%1.8؛ بفاصل ثقة مقداره %95: 1.2 إلى 2.7) ضعف المعدل بين البنات (%0.7؛ بفاصل ثقة مقداره %95: 0.4 إلى 1.3). ارتبطت نتائج اختبار الأجسام المضادة السلبية للتيتانوس، والإقامة في الريف، والأسر الأكثر فقرًا، بارتفاع معدل انتشار إيجابية مستضد HBsAg. لم نلحظ أي اختلاف في معدل الانتشار حسب العمر. كانت احتمالات إيجابية إصابة الأطفال بمستضد HBsAg أعلى إذا كانوا يعيشون مع فرد بالغ أو أكثر من أفراد الأسرة المصابين بالتهاب الكبد الوبائي ب (نسبة الاحتمالات المعدلة: 2.3؛ ‏بفاصل ثقة مقداره %95: 0.7 إلى 7.8)، وخاصةً إذا كانت الأم مصابة بالتهاب الكبد الوبائي ب (نسبة الاحتمالات المعدلة: 7.2؛ ‏بفاصل ثقة مقداره %95: 1.6 إلى 32.3). والجدير بالذكر أن ما يقرب من ثلثي (36/51) الأطفال المصابين بالتهاب الكبد الوبائي ب كانت أمهاتهم غير مصابات بالتهاب الكبد الوبائي ب.

الاستنتاج يسلط البحث الضوء على أهمية تقديرات الانتشار على المستوى دون الوطني في الدول الكبيرة مثل جمهورية الكونغو الديمقراطية، وقد حددنا مناطق قد تستفيد من تحسين استراتيجيات تقديم لقاحات الأطفال، وجهود الوقاية المجتمعية من فيروس التهاب الكبد الوبائي ب.

## 摘要

**目的:**

描述儿童乙肝病毒 (HBV) 流行病学的特征,为非洲人口最多的国家之一刚果民主共和国提供信息,以便其开展病毒消除工作。

**方法:**

通过运用最近(2013-2014 年)开展的具有全国代表性的人口和保健调查,我们分析了干血斑上的乙肝表面抗原 (HBsAg) 以及 6-59 个月大儿童的相关调查数据。我们从全国层面、从地区层面和按照各潜在感染相关因素估算了 HBsAg 阳性患病率。我们在总体上评估了 HBsAg 阳性患病率的空间变化,并按年龄、性别和疫苗接种状况分别进行了评估。

**结果:**

通过研究具有代表性的 5,773 名儿童的数据,我们发现全国 HBsAg 阳性患病率为 1.3%【73/5773;95% 置信区间 (CI):0.9 至 1.7】,各省市的患病率从金沙萨市的 0.0% 到南乌班吉的 5.6% 不等。男孩的患病率(1.8%;95% CI:1.2 至 2.7)是女孩(0.7%;95% CI:0.4 至 1.3)的两倍。破伤风抗体检测结果呈阴性、农村地区和较贫困家庭的儿童的 HBsAg 阳性患病率较高。据我们观察,不同年龄段的患病率并无差别。与一名或多名 HBsAg 呈阳性的成年家人【特别是在其母亲 HBsAg 呈阳性的情况下(比值比 (OR):7.2;95% CI:1.6 至 32.3)】共同生活的儿童出现 HBsAg 阳性的几率更高【OR:2.3;95% CI:0.7 至 7.8】。值得注意的是,近三分之二 (36/51) HBsAg 呈阳性的儿童,其母亲的 HBsAg 却是呈阴性。

**结论:**

我们的调查凸显了在刚果民主共和国等大国估算地方患病率的重要性,而且我们已确定可能会因改进儿童疫苗接种实施策略和社区开展 HBV 预防工作而受益的地区。

## Резюме

**Цель:**

Охарактеризовать эпидемиологию вируса гепатита В (HBV) у детей для обеспечения информационной поддержки усилий по устранению этого заболевания в одной из наиболее населенных стран Африки – Демократической Республике Конго.

**Методы:**

Авторы использовали результаты последнего национального репрезентативного исследования демографии и здоровья населения (2013–2014 гг.) и проанализировали данные о наличии поверхностного антигена вируса гепатита В (HBsAg) в высушенных образцах крови. Анализу также подверглись сопутствующие данные исследования детей в возрасте от 6 до 59 месяцев. Была выполнена приблизительная оценка распространенности положительного результата по HBsAg в национальном и региональном масштабах, а также рассчитано распределение этого показателя по потенциальным коррелятам инфекции. Авторы также оценили пространственные вариации распространенности положительного результата по HBsAg в целом и с распределением по возрасту, полу и статусу вакцинирования.

**Результаты:**

Судя по данным для 5773 детей, наблюдаемая распространенность положительного результата теста на HBsAg в национальном масштабе составляет 1,3% (73 из 5773, 95%-й доверительный интервал, ДИ: 0,9–1,7), в диапазоне от 0% в Киншасе до 5,6% в Южном Убанги. Распространенность среди мальчиков (1,8%; 95%-й ДИ: 1,2–1,7) вдвое выше, чем среди девочек (0,7%; 95%-й ДИ: 0,4–1,3). Более высокая распространенность положительной реакции на HBsAg ассоциировалась с отрицательным результатом тестирования на антитела к столбняку, проживанием в сельской местности и более низким уровнем дохода семьи. Различий в распространенности инфекции по возрасту не отмечалось. Более высокая вероятность получить положительный результат тестирования на HBsAg наблюдалась у детей, проживающих вместе с одним или несколькими взрослыми членами семьи с HBsAg-положительным статусом (отношение шансов, ОШ: 2,3; 95%-й ДИ: 0,7–7,8), в частности с положительной по HBsAg матерью (ОШ: 7,2; 95%-й ДИ: 1,6–32,3). Примечательно, что матери у почти двух третей (36 из 51) положительных по HBsAg детей имели отрицательный результат по HBsAg.

**Вывод:**

Данное исследование подчеркивает важность оценки распространенности заболевания на субнациональном уровне в таких крупных странах, как Демократическая Республика Конго. Авторы также выявили регионы, в отношении которых целесообразно рассмотреть внедрение более эффективных стратегий вакцинирования детей и оптимизации усилий по профилактике HBV на уровне сообществ.

## Introduction

An estimated 6.2 million children younger than 5 years are infected with hepatitis B virus (HBV) globally, two thirds of whom live in Africa.^1^ Prevention of HBV acquisition in children younger than 5 years is critical, as they have a substantially higher risk of developing chronic HBV infection following exposure (> 90%) compared with adults (< 10%).^2^ HBV prevention in children is focused on the prevention of perinatal transmission, with hepatitis B surface antigen (HBsAg) testing during pregnancy; antenatal antiviral prophylaxis administered to mothers with high serum HBV DNA (deoxyribonucleic acid) levels (≥ 200 000 IU/mL); HBV birth-dose vaccination and post-exposure prophylaxis with HBV immunoglobulin where available; along with a triple-dose hepatitis B vaccine series given in infancy.^3^ Although these interventions are highly effective in preventing perinatal transmission,^4^^–^^6^ they are not fully implemented in many parts of the African region.^7^ Further, epidemiological studies from several African countries suggest that early horizontal transmission may contribute more to high HBV prevalence in children than in other regions globally;^8^^–^^12^ however, the viral, immunological and sociocultural drivers of these transmission patterns remain poorly understood in this region. 

HBV prevalence is not well quantified in the Democratic Republic of the Congo, the third most populous African country.^13^^–^^15^ The estimates that are available for central Africa broadly suggest an HBsAg-positivity prevalence of 10–14% but also that fewer than 1% of infections are diagnosed.^16^ Because of the lack of nationally representative data in the region, central African countries contribute the largest uncertainty to global estimates.^17^ Current HBV prevention approaches in the country include triple-dose vaccination at the ages of 6, 10 and 14 weeks, first introduced in the national infant immunization program in 2007 and given as part of the pentavalent vaccine (diphtheria–pertussis–tetanus [DPT], triple-dose hepatitis B and *Haemophilus influenzae* B).^18^^,^^19^ Triple-dose vaccination coverage among those aged 1 year has stagnated, with estimates beginning at 65% in 2007, peaking at 73% in 2014 and 2019, and decreasing to 60% in 2023, the most recent year available.^20^ Antenatal HBsAg testing and birth-dose vaccination have yet to be implemented nationally, and HBV immunoglobulin is unavailable. Blood products are tested using rapid HBsAg tests, but test kit supply remains inconsistent, particularly in rural areas,^21^ and HBV transmission from transfusions remains a concern.^22^

We previously analysed dried blood spot samples from a subset of 277 infants and young children (age 6–59 months) in the most recent available (2013–2014) DHS for the Democratic Republic of the Congo, finding an HBV prevalence of 2.2%;^23^ however, evaluation of spatial variation and infection correlates was precluded in this previous study by its limited sample size. In the current study, we analysed all remaining samples (> 5600) from the 2013–2014 DHS to more fully characterize HBV epidemiology in this setting. Collected 6 years after the triple-dose vaccine was introduced in the national infant immunization programme, these data can provide some insights on its impact through evaluation of HBV prevalence by age in children younger than 5 years. As the triple-dose vaccine is one of the few available interventions in the country and coverage has not improved in the last decade, our analysis identifies populations for focused interventions, critical for informing elimination efforts in this setting.

## Methods

### Study population and design

Our characterization of HBV epidemiology among infants and young children aged 6–59 months had three primary objectives: (i) estimation of childhood HBsAg-positivity prevalence nationally, provincially and by subgroup, including by age and sex; (ii) assessment of correlates of HBsAg-positivity prevalence among children; and (iii) estimation of the odds of infection among children sharing a household with an HBV-infected adult (any adult and their mother specifically, in two different analyses) through a nested case–control study. We used survey data and dried blood spot samples from the 2013–2014 DHS, a population-representative survey that included sample collection in a random subset of 50% of households (online repository).^24^ Detailed survey method is described in the 2013–2014 DHS final report for the country.^25^

For the first and second study objectives, we included all children for which a dried blood spot sample was available. We chose a nested case–control design for the third objective, as HBV transmission from mothers or other individuals within households is common; however, evaluating all adult samples was impracticable. We defined case households as those with exactly one child aged 6–59 months who was HBsAg positive in our biospecimen analysis; these children comprised the case population. Control households were sampled in a 6:1 ratio (control:case households) from households in which all children tested were HBsAg negative; these children (possibly more than one per household) were the controls. As the sampling frame of control households excluded those with HBsAg-negative children who lived with a HBsAg-positive child, we excluded HBsAg-positive children who lived with another HBsAg-positive child from cases (three households total). Within these case and control households, we evaluated adult household members with dried blood spot samples for HBV infection as a potential correlate of infection for the case versus control children.

### Biospecimen analysis

We determined HBV status by eluting dried blood spot samples and analysing HBsAg presence on the ARCHITECT platform (Abbott Laboratories, Abbott Park, United States of America; online repository).^24^ The ARCHITECT instrument uses a signal-to-cutoff ratio in determining HBsAg positivity. To reduce the risk of false-positive results, we employed a two-step approach: (i) any sample with a ratio of more than 1 was retested automatically using the same eluant (technical replicate); and (ii) if sufficient sample remaining, the assay was repeated using a fresh 6-mm punch (biological replicate) for any positive sample with a ratio of 1:100 or with an adjacent positive on the elution plate. We considered samples with insufficient material for repeat testing positive in the primary analysis if the average ratio of both technical replicates was 5 or greater, and negative otherwise. We considered all other samples positive if both positive biological replicates (four total technical replicates) were positive (ratio ≥ 5) and negative otherwise. We conducted sensitivity analyses with different positivity cut-offs (ratios of 1, 2 and 100).

### HBsAg prevalence and correlates analysis

For representative estimates of HBsAg positivity prevalence, we applied two types of weights. To account for the probability of selection into the survey, we used weights available through the DHS Program. To account for missing or exhausted samples among those selected to provide biospecimens, we followed an established approach to calculate propensity score weights using inverse probability of sampling (treatment) weighting.^26^^,^^27^ We identified attributes that could be associated with the outcome (HBsAg status) or missing or exhausted samples (online repository).^24^ These variables were included as independent predictors in a binomial logistic regression model in which having an HBsAg result (yes or no) was the dependent variable. We calculated predicted probabilities from this model to obtain propensity scores, the inverse of which formed the weights. We stabilized the inverse propensity score weights by dividing each weight by the sum of weights in the group with samples. We multiplied the stabilized weights by the DHS weights to create final survey weights in the analysis.

We identified variables available from the DHS to assess as potential correlates of HBV infection, including caretaker-reported age, sex and household wealth; province, rurality and location; anaemia, *Plasmodium falciparum* malaria infection, tetanus serology results (a proxy for pentavalent vaccination) and growth stunting; and caretaker reporting of injections received in the last 12 months (e.g. antibiotics and antimalarial medications), use of a new and unopened needle and/or syringe for the last injection (if one reported in the past year) and justification of violence towards women in the household in at least one circumstance on a provided list (online repository).^24^

We described the distribution of the overall population according to each potential correlate of interest, and estimated HBsAg-positivity prevalence and Wald-type 95% CIs within each strata. We estimated prevalence differences and associated Wald-type 95% CIs in HBsAg positivity across correlate strata.

### Spatial analysis

We characterized spatial variation in HBsAg-positivity at the DHS cluster level and at the province level. Global positioning system (GPS) locations were available through the DHS Program for DHS clusters; for clusters with missing location, we used province designation to randomly select latitude and longitude coordinates within the province to impute coordinates. We calculated prevalence of HBsAg positivity within each DHS cluster. We also analysed choropleth maps of weighted HBsAg-positivity prevalence at the province level, overall and by age, sex and triple-dose vaccination proxies (tetanus antibody-positive versus antibody-negative, and reported receipt of any DPT vaccine dose versus none). To inform focused public health action, we mapped province-level prevalence differences by these same variables.

### Nested case–control analysis

We used logistic regression to calculate sampling-weighted, unadjusted ORs comparing HBsAg positivity in case versus control children according to exposure to: (i) at least one HBsAg-positive adult household member of any type, and (ii) an HBsAg-positive mother. As with our assessment of HBV correlates in the full study population via unadjusted prevalence differences, the unadjusted estimation of ORs was primary in our nested case–control analysis because of the descriptive (i.e. non-causal) nature of our study. In this component of the study, our objective was to descriptively assess whether odds of infection among children in households with HBsAg-positive adults was higher than in households without HBsAg-positive adults, regardless of whether this association was due to a causal effect (i.e. direct transmission from adult to child) or simply co-occurrence as a result of more complex (but unmeasurable) transmission dynamics. We also explored adjustment by age (in months), sex and vaccination status (using reported DPT vaccination and tetanus serology) in supplementary analyses to broadly illustrate some potential interrelationships among HBV and these variables in the study sample.

### Statistical analysis

All data were imported and analysed in R version 4.3.3 (R Core Team, Vienna, Austria) using the tidyverse, survey and srvr packages. We conducted spatial analysis using the sf package. The R code that we wrote for this analysis is publicly available.^28^

### Ethics

This study was approved by the Institutional Review Board at the University of North Carolina, United States of America, and the Ethics Committee at Kinshasa School of Public Health, Democratic Republic of the Congo.

## Results

### Study population

We analysed 5679 samples, representing 5773 (weighted) children 6–59 months of age (online repository).^24^ The median age among children was 32 months (interquartile range: 18–46) and their households had a median of seven residents. Most children (4053/5773; 70.2%) were from rural areas, and 44.5% (2570/5773) resided in households in the lower two wealth quintiles (Table 1). Similar numbers of boys and girls were studied. Of the study population, 1629 (28.3%) had polymerase chain reaction-confirmed *P. falciparum* infections, and about one third (2092; 36.2%) had detectable tetanus antibodies. Nutritional status was poor overall: almost half (2564; 44.4%) were moderately-to-severely stunted and about one third (1994; 34.5%) had moderate-to-severe anaemia. About half of the children (3057; 53.0%) had documented or reported DPT vaccine completion, and 17.9% (1034) had not initiated the series; DPT vaccination status was distributed similarly across age groups (online repository).^24^ Almost one third of children (1799; 31.2%) had received at least one injection in the last year, and for 6.9% (125/1799) of those children, a used needle or syringe was used in their last injection. Approximately two thirds of children (3955/5773; 68.5%) lived in a household in which physical violence towards a woman was reported as justified. These distributions of demographics of children evaluated for HBV were similar to those of all children selected for DHS biospecimen collection (online repository),^24^ and similar to those included in the nested case–control study (Table 1).

**Table 1:** Study population characteristics and hepatitis B virus prevalence among children aged 6–59 months in the Democratic Republic of the Congo, 2013–2014

Characteristic	No. (%)^ a^				Prevalence (95% CI)^a^	
	Sampled population (*n* = 5773)	HBsAg-positive children (*n* = 73)	Nested case–control study		HBsAg positivity	Difference per 100 children
			Cases (*n* = 58)	Controls (*n* = 571)		
**Age, months**						
6–11	683 (11.8)	8 (11.3)	8 (13.7)	91 (15.9)	1.2 (0.6 to 2.5)	Reference
12–23	1235 (21.4)	13 (17.2)	10 (17.8)	130 (22.7)	1.0 (0.4 to 2.7)	−0.2 (−1.5 to 1.1)
24–35	1297 (22.5)	17 (23.9)	14 (24.8)	131 (22.9)	1.3 (0.8 to 2.2)	0.1 (−1.0 to 1.3)
36–47	1260 (21.8)	20 (27.7)	14 (23.4)	125 (21.9)	1.6 (0.9 to 3.0)	0.4 (−0.9 to 1.7)
48–59	1298 (22.5)	15 (20.0)	12 (20.3)	95 (16.6)	1.1 (0.6 to 2.1)	−0.1 (−1.2 to 1.0)
**Sex**						
Male	2917 (50.5)	53 (72.7)	42 (72.6)	290 (50.8)	1.8 (1.2 to 2.7)	1.1 (0.2 to 2.0)
Female	2855 (49.5)	20 (27.3)	16 (27.4)	281 (49.2)	0.7 (0.4 to 1.3)	Reference
**Relationship to household head**						
Child	4520 (78.3)	60 (81.8)	50 (85.5)	453 (79.3)	1.3 (0.9 to 1.9)	Reference
Grandchild	901 (15,6)	8 (11.0)	5 (8.9)	79 (13.8)	0.9 (0.4 to 2.0)	−0.4 (−1.4 to 0.5)
Other	352 (6.1)	5 (7.1)	3 (5.6)	39 (6.8)	1.5 (0.6 to 3.3)	0.2 (−1.2 to 1.5)
**Location of residence**						
Rural countryside	4053 (70.2)	60 (82.3)	47 (81.1)	409 (71.7)	1.5 (1.0 to 2.1)	0.6 (−0.3 to 1.6)
Provincial capital	958 (16.6)	8 (11.1)	8 (14.0)	109 (19.1)	0.8 (0.3 to 2.1)	Referent
Town	623 (10.8)	4 (5.3)	3 (4.9)	44 (7.7)	0.6 (0.3 to 1.5)	−0.2 (−1.2 to 0.7)
Small city	139 (2.4)	1 (1.3)	0 (0.0)	9 (1.5)	0.7 (0.1 to 6.1)	−0.1 (−1.7 to 1.4)
**Wealth^b^ **						
Poorest	1310 (22.7)	25 (34.0)	21 (35.6)	115 (20.2)	1.9 (1.1 to 3.1)	Reference
Poorer	1260 (21.8)	19 (26.4)	15 (26.7)	128 (22.5)	1.5 (0.8 to 3.0)	−0.4 (−1.8 to 1.1)
Middle	1159 (20.1)	15 (20.6)	12 (21.4)	102 (17.9)	1.3 (0.7 to 2.4)	−0.6 (−1.8 to 0.6)
Richer	1149 (19.9)	9 (11.6)	8 (13.7)	143 (25.0)	0.7 (0.3 to 1.6)	−1.2 (−2.3 to −0.1)
Richest	895 (15.5)	5 (7.4)	2 (2.6)	82 (14.4)	0.6 (0.2 to 2.1)	−1.3 (−2.6 to 0.0)
***P. falciparum* malaria infection^c^**						
Negative	4137 (71.7)	48 (66.2)	41 (71.0)	444 (77.8)	1.2 (0.8 to 1.8)	Reference
Positive	1629 (28.3)	25 (33.8)	17 (29.0)	127 (22.2)	1.5 (1.0 to 2.2)	0.3 (−0.4 to 1.1)
**Stunting^d^**						
Moderate to severe	2564 (44.4)	34 (46.8)	29 (50.3)	269 (47.0)	1.3 (0.9 to 2.0)	Reference
None	2994 (51.9)	36 (49.6)	27 (46.8)	272 (47.6)	1.2 (0.8 to 2.0)	−0.1 (−0.9 to 0.6)
Missing	215 (3.7)	3 (3.5)	2 (2.9)	30 (5.3)	NA	NA
**Anaemia^e^**						
Moderate to severe	1994 (34.5)	30 (41.2)	27 (46.5)	186 (32.5)	1.1 (0.8 to 1.7)	Reference
Mild to none	3773 (65.4)	43 (58.8)	31 (53.5)	385 (67.5)	1.5 (0.9 to 2.4)	0.4 (−0.5 to 1.2)
Missing	6 (0.1)	0 (0.0)	0 (0.0)	0 (0.0	NA	NA
**Positive for tetanus antibodies^f^**						
Reactive	2092 (36.2)	15 (20.6)	13 (22.3)	254 (44.4)	0.7 (0.4 to 1.4)	−0.8 (−1.6 to −0.1)
Nonreactive	3610 (62.5)	56 (77.0)	43 (74.7)	317 (55.6)	1.6 (1.1 to 2.3)	Reference
Indeterminant	71 (1.2)	2 (2.4)	2 (3.0)	0 (0.0	2.5 (0.3 to 17.1)	0.9 (−4.0 to 5.8)
**Diphtheria–pertussis–tetanus vaccination^g^ **						
Series completed	3057 (53.0)	31 (42.7)	26 (45.3)	349 (61.1)	1.0 (0.6 to 1.7)	−1.2 (−2.6 to 0.1)
Series incomplete	949 (16.4)	6 (7.6)	5 (8.8)	89 (15.6)	0.6 (0.3 to 1.3)	−1.7 (−3.0 to −0.3)
No doses received	1034 (17.9)	23 (31.8)	18 (31.7)	78 (13.7)	2.2 (1.3 to 3.9)	Reference
Not available	732 (12.7)	13 (17.9)	8 (14.1)	55 (9.6)	NA	NA
**Injections in last year^h^ **						
1–12	1436 (24.9)	14 (19.2)	10 (17.4)	188 (32.9)	1.0 (0.5 to 1.8)	−0.3 (−1.0 to 0.5)
13–24	223 (3.9)	3 (4.6)	2 (3.4)	10 (1.7)	1.5 (0.6 to 3.9)	0.3 (−1.3 to 1.8)
≥ 25	141 (2.4)	3 (3.7)	3 (4.6)	10 (1.7)	1.9 (0.5 to 7.5)	0.7 (−2.0 to 3.4)
None	3254 (56.4)	40 (54.7)	35 (60.5)	309 (54.1)	1.2 (0.8 to 2.0)	Reference
Missing	719 (12.5)	13 (17.9)	8 (14.1)	55 (9.6)	NA	NA
**Physical violence towards wife^i^**						
Justified	3955 (68.5)	52 (71.2)	44 (75.4)	415 (72.7)	1.3 (0.9 to 2.0)	Reference
Never justified	1100 (19.1)	8 (10.9)	6 (10.4)	101 (17,7)	0.7 (0.4 to 1.4)	−0.6 (−1.3 to 0.1)
Missing	718 (12.4)	13 (17.9)	8 (14.1)	55 (9.6)	NA	NA

DHS: Demographic and Health Survey; HBsAg: hepatitis B surface antigen; NA: not applicable.

^a^ The numbers of children in columns 2–5 were weighted according to DHS sampling weights and inverse propensity score weights to account for missing or exhausted samples. Although rounded to whole numbers for inclusion in the table, the unrounded counts were used to calculate the percentages. The sum of rounded percentages may therefore differ from 100.0 by a few decimal points.

^b^ As defined by the DHS Program for the full population of the Democratic Republic of the Congo.

^c^ *Plasmodium falciparum* infection determined by polymerase chain reaction targeting the *Pfldh* gene using DHS dried blood spots.^29^

^d^ Stunting was assessed using standardized height-for-age scores, with a score of < 2 standard deviations indicative of moderate-to-severe stunting.

^e^ Anaemia was assessed with point-of-care haemoglobin testing with a correction for altitude. Mild, moderate and severe anaemia was defined as a haemoglobin level of 10.0–10.9, 7.0–9.9 and < 7.0 g/dL, respectively.

^f^ Tetanus antibody results, produced using an ELISA-based assay, were available through DHS Program.^30^

^g^ As of 2007, the diphtheria–pertussis–tetanus (DPT) series includes hepatitis B vaccination.

^h^ According to the caretaker, 125 of the 1799 (6.9%) who received injections in the last year received their injection with an open or previously used syringe.

^i^ Determined as a “yes” response to questions about whether beating one’s wife is justified (wife goes out without telling husband; wife neglects children; wife argues with husband; wife refuses sex with husband; wife burns food). These questions are separate from the DHS Domestic Violence Module, the questions from which were put to a different subset of DHS households.

### HBsAg-positivity prevalence

We observed an overall HBsAg-positivity prevalence of 1.3% (73/5773; 95% CI: 0.9 to 1.7) (Table 1), ranging from 1.2% to 1.7% in sensitivity analyses (online repository).^24^ HBsAg-positivity prevalence was 1.8% (53/2917; 95% CI: 1.2 to 2.7) in boys and 0.7% in girls (20/2856; 95% CI: 0.4 to 1.3), corresponding to a prevalence difference of 1.1 cases (95% CI: 0.2 to 2.0) per 100 children. HBsAg-positivity prevalence did not differ appreciably by age, ranging from 1.0% (13/1235) in those aged 12–23 months to 1.6% (20/1260) among those aged 36–47 months (Table 1; online repository).^24^ Prevalence among children in rural areas (60/4053; 1.5%; 95% CI: 1.0 to 2.1) was nearly double that of children from non-rural areas (13/1720; 0.8%; 95% CI: 0.4 to 1.5). Prevalence decreased with increasing household wealth, from 1.9% (25/1310; 95% CI: 1.1 to 3.1) in the poorest wealth quintile to 0.6% (5/895; 95% CI: 0.2 to 2.1) in the richest wealth quintile. HBsAg-positivity prevalence among children infected with *P. falciparum* was 1.5% (25/1629; 95% CI: 1.0 to 2.2), compared with 1.2% (48/4137; 95% CI: 0.8 to 1.8) among those uninfected with *P. falciparum*.

Children with detectable tetanus antibodies had 0.8 fewer (95% CI: −1.6 to −0.1) HBsAg-positive cases per 100 children compared with those without detectable antibodies. DPT vaccination reported by caretakers was associated with lower HBsAg positivity, with children reported to have initiated the series (1–2 doses) having 1.7 fewer (95% CI: −3.0 to −0.3) infections per 100 compared with those who had not received any doses (Table 1; online repository).^24^ Children in households in which physical violence towards women was never justified had 0.6 fewer (95% CI: −1.3 to 0.1) HBsAg-positive cases per 100 children compared with households in which violence was reported as justified. We observed no significant association between HBsAg positivity and anaemia, growth stunting or receiving injections in the past 12 months.

### Spatial variation in HBsAg-positivity prevalence

**Fig. 1:**
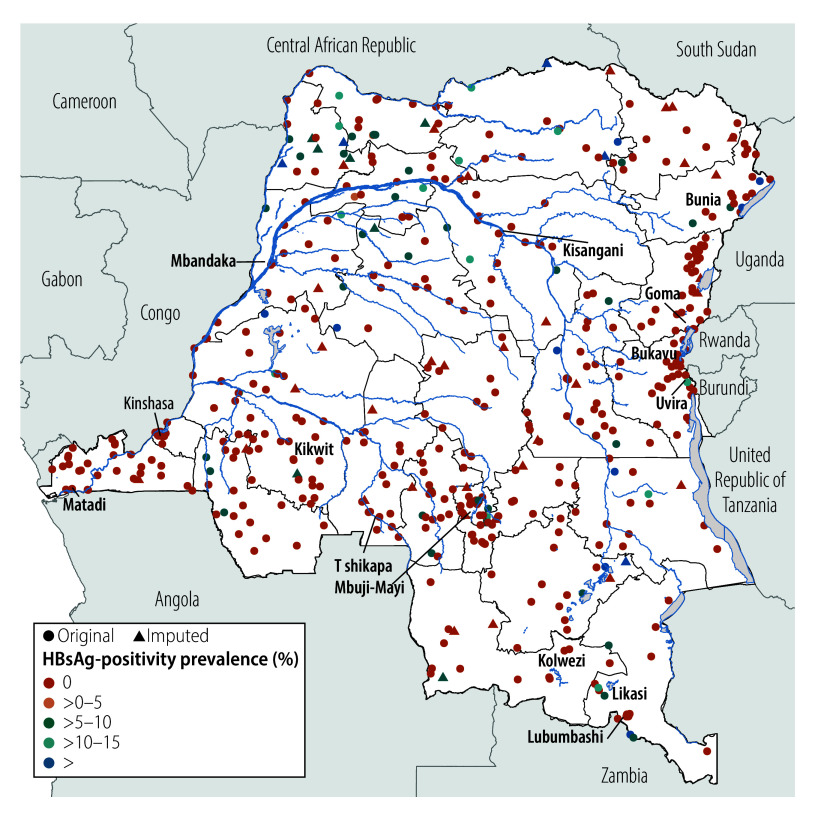
Geographic distribution of the prevalence of HBsAg positivity in children aged 6–59 months, by DHS cluster location, Democratic Republic of the Congo, 2013–2014

DHS: Demographic and Health Survey; HBsAg: hepatitis B surface antigen.

**Fig. 2:**
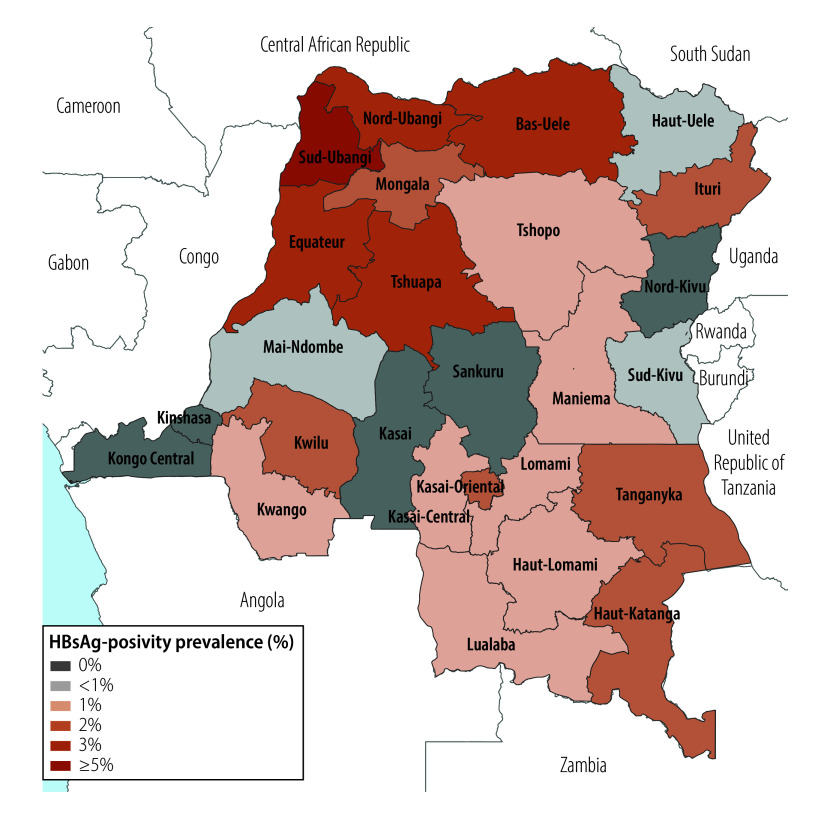
Geographic distribution of the prevalence of HBsAg positivity in children aged 6–59 months, by province, Democratic Republic of the Congo, 2013–2014

**Fig. 3:**
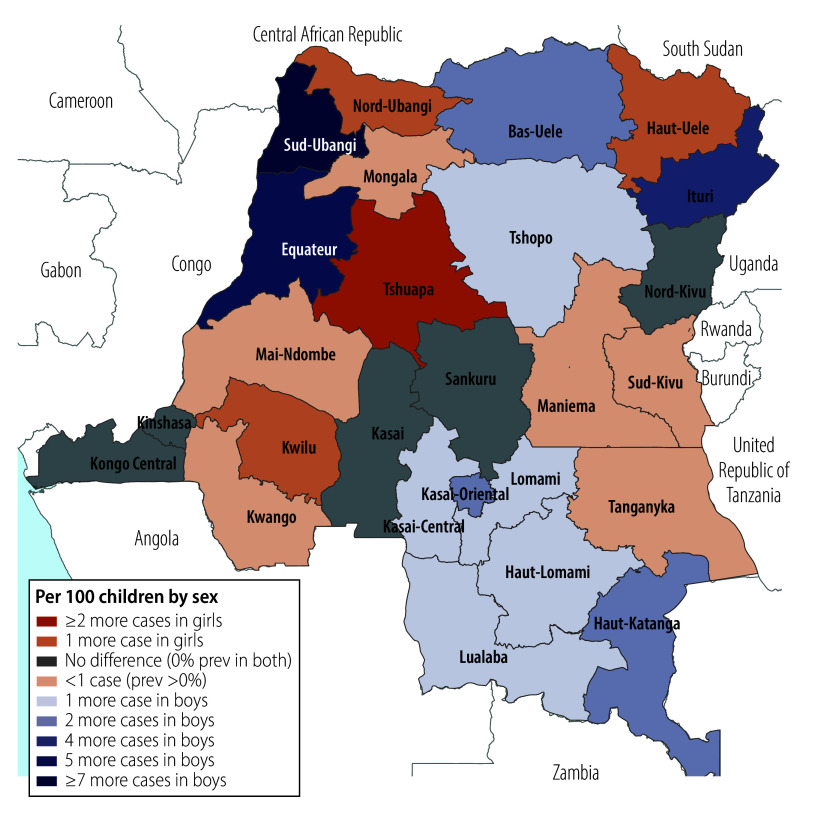
Geographic distribution of province-level prevalence differences per 100 children in HBsAg positivity in children aged 6–59 months, by sex, Democratic Republic of the Congo, 2013–2014

**Fig. 4:**
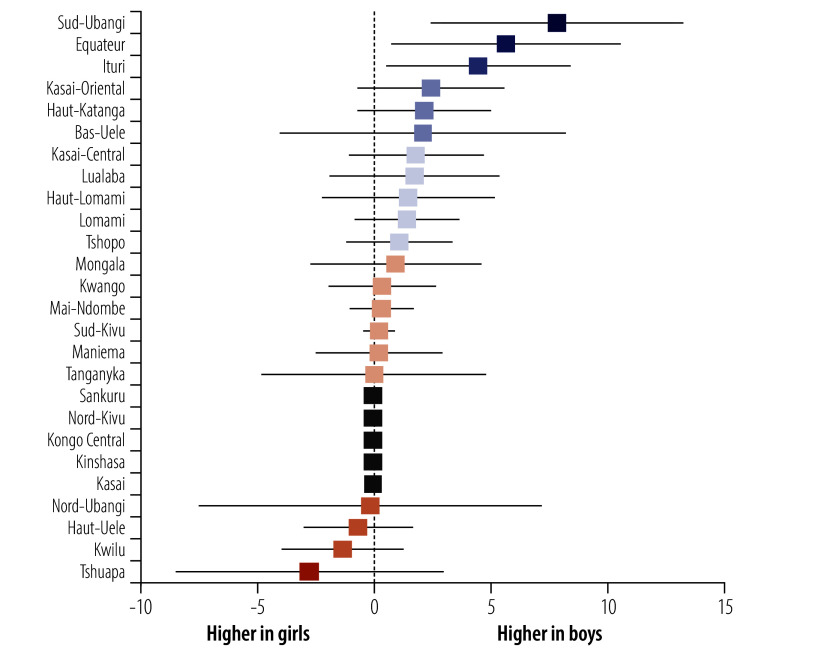
Province-level prevalence differences per 100 children in HBsAg positivity in children aged 6–59 months, by sex, Democratic Republic of the Congo, 2013–2014

**Fig. 5:**
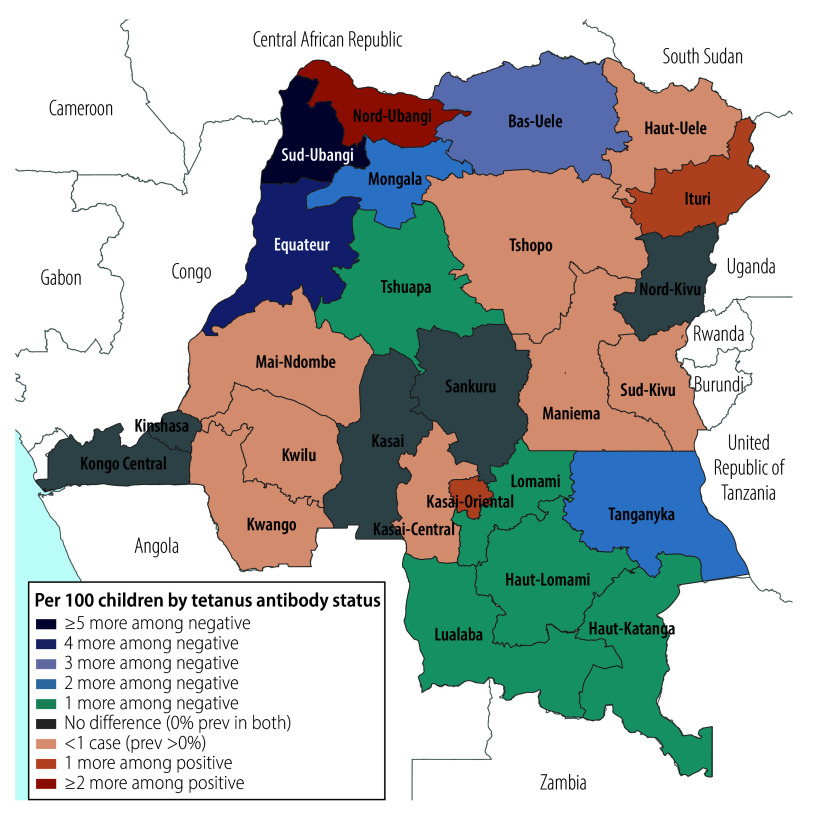
Geographic distribution of province-level prevalence differences per 100 children in HBsAg positivity in children aged 6–59 months by tetanus antibody status, Democratic Republic of the Congo, 2013–2014

**Fig. 6:**
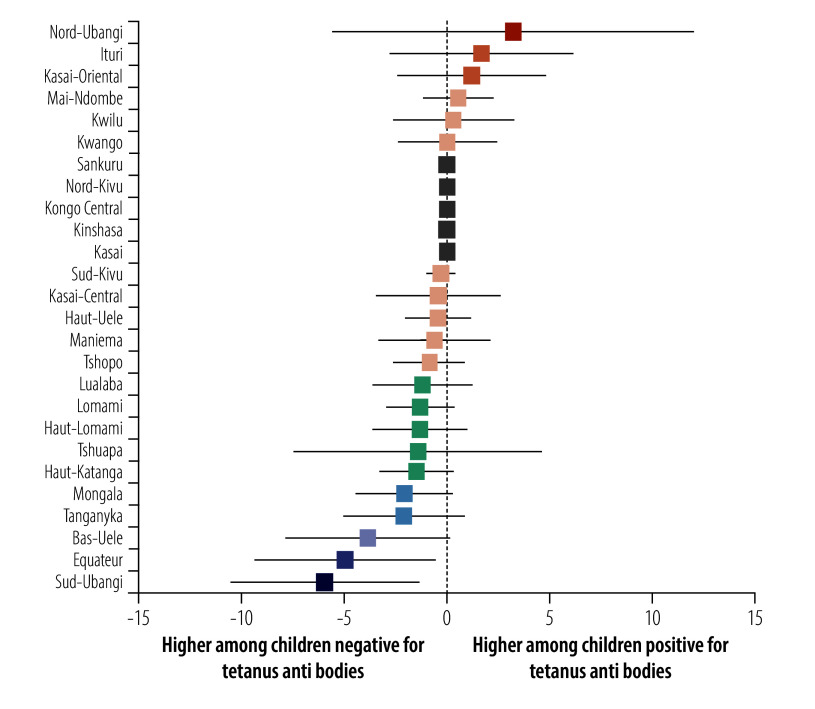
Province-level prevalence differences per 100 children in HBsAg positivity in children aged 6–59 months by tetanus antibody status, Democratic Republic of the Congo, 2013–2014

We observed a weighted HBsAg-positivity prevalence of greater than 10% in 30 DHS clusters, representing 750–900 households (Fig. 1). We observed substantial provincial variation, from 0% in several provinces (95% CI: 0 to 0) to 5.6% (95% CI: 2.6 to 11.8) in the northwestern Sud-Ubangi province (Fig. 2; online repository).^24^ We identified at least one HBsAg-positive child in most (21 of 26) provinces. Twelve provinces had higher HBsAg-positivity prevalence among boys compared with girls, with the largest differences in Sud-Ubangi, Équateur and Ituri; two provinces (Tshuapa and Kwilu) had higher HBsAg-positivity prevalence among girls compared with boys (Fig. 3; Fig. 4; online repository).^24^ In 10 provinces, children who were negative for tetanus antibodies had higher HBsAg positivity compared with children who were seropositive, with the largest magnitude in Sud-Ubangi (prevalence difference: 5.9 per 100 children, 95% CI: 1.4–10.5) (Fig. 5; Fig. 6; online repository).^24^ DPT vaccination resulted in similar directionality of association as that observed between tetanus seropositivity and HBsAg status in most provinces, but the opposite effect was observed in two provinces (Lualaba and Tanganyka, both in the Katanga region), in which any documented or reported DPT doses was associated with higher HBsAg-positivity prevalence (online repository).^24^ In no province did HBsAg positivity consistently decrease with decreasing age group (online repository).^24^

HBsAg: hepatitis B surface antigen.

HBsAg: hepatitis B surface antigen.

HBsAg: hepatitis B surface antigen.

HBsAg: hepatitis B surface antigen.

HBsAg: hepatitis B surface antigen.

### Nested case–control study

In our nested case–control study, we observed that two-thirds (38/58; 65.5%) of HBsAg-positive children had no HBsAg-positive household members, and nearly two-thirds (36/58; 62.1%) of HBsAg-positive children had a HBsAg-negative mother. HBsAg positivity was more likely to be observed (OR: 2.3; 95% CI: 0.7 to 7.8) in children living with an HBsAg-positive adult than in children not living with an HBsAg-positive adult (Table 2). Children living with a mother who was HBsAg positive were much more likely (OR: 7.2; 95% CI: 1.6 to 32.3) to demonstrate HBsAg positivity compared with those living with an HBsAg-negative mother (Table 2). Exploratory adjustment for age, sex and evidence of vaccination resulted in a shift towards the null and loss of precision in both analyses (online repository).^24^

**Table 2:** Nested case–control study evaluating exposure of children aged 6–59 months to an HBsAg-infected adult household member, Democratic Republic of the Congo, 2013–2014

Exposure level	No. cases (HBsAg-positive children)^a^	No. controls (HBsAg-negative children)^a^	Unadjusted OR (95% CI)^a^
**No. HBsAg-positive adult household members**			
≥ 1	20	103	2.3 (0.7 to 7.8)
0	38	468	Reference
**HBsAg status of mother**			
Positive	15	27	7.2 (1.6 to 32.3)
Negative	36	460	Reference
Information unavailable	7	84	NA

CI: confidence interval; HBsAg: hepatitis B surface antigen; NA: not applicable; OR: odds ratio.

^a^ Weighted by Demographic and Health Survey sampling weights and inverse propensity score weights accounting for missing and exhausted samples.

## Discussion

In the largest national survey of HBV among children and household contacts in the Democratic Republic of the Congo, we found that childhood HBsAg prevalence was 10–60 times the global target of 0.1%. 

We observed a sex-based difference in prevalence, which has been well documented in HBV epidemiology.^31^ Understanding local causes is important for designing effective interventions. Circumcision with insufficiently sanitized instruments^32^ and other traditional sex-specific practices (e.g. female genital mutilation)^33^ could contribute to differential infection risk by sex. These practices likely vary across this culturally diverse country, but are not well characterized in national surveys.^34^^,^^35^ Higher prevalence among boys could also be explained by poorer hepatitis B surface antibody response from vaccination among boys compared with girls, observed previously in Senegal.^36^ HBV serology to evaluate protection or past exposure remains difficult to study in remote settings because of the limitations of assay performance on cold-chain-independent samples such as dried blood spots,^37^ and is an area for future development. The explanations hypothesized here for the observed prevalence difference by sex relate to modes of horizontal transmission, highlighting the need to consider prevention of horizontal HBV transmission in this context.^11^

Reactive tetanus antibodies and reported DPT vaccination, proxies for triple-dose hepatitis B vaccination, were both associated with lower HBsAg positivity. We observed substantial regional variation in this association, with the protective effect of vaccination against HBsAg positivity observed in only half of the provinces. Although tetanus serology and caretaker-reported vaccination are imperfect measurements of HBV vaccination, our results suggest that follow-up evaluation of pentavalent vaccine storage, distribution and administration in these provinces may be worthwhile, as triple-dose and DPT vaccine coverage have declined in the decade since this survey was conducted.^38^^,^^39^

We did not observe a difference in HBsAg positivity between children infected and uninfected with *P. falciparum* malaria, but our study was not optimally designed to interrogate the mechanisms through which an acute infection such as malaria could influence HBV infection. It is important to evaluate interactions between malaria, HBV infection and clinical care, particularly as the first licensed malaria vaccines, which contain the HBsAg protein as an adjuvant, will soon be implemented in the Democratic Republic of the Congo.^40^ Malaria vaccination has been shown to boost hepatitis B surface antibody levels,^41^ but the impact on HBV prevalence in the population remains unknown.

Living with an HBsAg-positive adult household member, particularly an HBsAg-positive mother, was associated with higher odds of HBsAg positivity in children. This result is unsurprising in a setting with no prevention of vertical transmission at birth, and minimal prevention of horizontal transmission except for the hepatitis B triple-dose vaccine (coverage only 60–70% in the past decade).^20^ The association with an HBsAg-positive mother is likely partly driven by vertical transmission at birth, but our findings that almost two-thirds of cases had HBsAg-negative mothers and two-thirds did not live with an HBsAg-positive household member suggest several additional potential mechanisms, for example: vertical transmission perinatally with subsequent recovery by mothers, or horizontal exposures from either outside the household or within the household from an unassessed individual. Horizontal HBV transmission in households and villages has been documented in settings across the continent, associated with the sharing of personal items and close contact with an infected individual.^10^^,^^42^^,^^43^ Family members are also commonly donors for blood transfusions, which are frequently needed for malaria-induced anaemia and often occur without sufficient infection screening as a result of test kit stockouts.^21^ These findings highlight the need for perinatal prevention through antenatal screening and birth-dose vaccination, as well as prevention of horizontal transmission through screening of blood products before transfusions, completion of vaccinations and sanitation of shared personal objects.

Our investigation had several limitations. First, our cross-sectional design cannot confirm directionality or timing of exposure; all associations must therefore be interpreted as descriptive, rather than causal. Second, these samples were analysed many years after collection, and it is possible that HBsAg protein degraded with time. However, our national prevalence estimates are slightly lower but generally consistent with those from earlier, smaller studies.^23^ Third, the survey was conducted during 2013–2014 and may not reflect the country’s current HBV infection landscape. Regardless, this analysis provides the most recent nationally representative estimates available in a setting that has yet to implement additional HBV prevention measures.

Our investigation has highlighted the importance of subnational prevalence estimates in large countries such as the Democratic Republic of the Congo, and we have identified regions that may benefit from improved childhood vaccination delivery strategies and community HBV prevention efforts. As new guidelines for HBV prevention are introduced,^44^ our analysis provides a timely investigation in a country in need of improved HBV control measures.
